# Editorial: Cognitive strategies in motor learning and rehabilitation

**DOI:** 10.3389/fneur.2025.1698611

**Published:** 2025-11-03

**Authors:** Daniel L. Eaves, Cosimo Costantino, Andrea Demeco, Giovanni Buccino

**Affiliations:** ^1^School of Biomedical, Nutritional and Sport Sciences, Faculty of Medical Sciences, Newcastle University, Newcastle, United Kingdom; ^2^University of Parma, Parma, Italy; ^3^Division of Neuroscience, San Raffaele Hospital (IRCCS), Milan, Italy; ^4^Università Vita-Salute San Raffaele, Milan, Italy

**Keywords:** motor imagery, mirror neurons, action observation, mental practice, neurorehabilitation, action simulation, motor learning and control

## Aims and context

Recent experimental evidence shows the motor system has evolved beyond its role as a simple executor of movement. Increasingly, it is viewed as an active participant in the perceptual-cognitive processes relating to action observation (AO), action understanding, motor imagery (MI), and verbal or symbolic representations of movement. This broader framework has led to the conceptualization of *action re-enactment*. This refers to the internal activation of motor-related neural structures in the absence of overt movement.

This Research Topic aims at providing a state-of-the-art overview of how cognitive strategies, such as AO and MI, are being deployed to understand and enhance motor performance across diverse populations. The contributions presented here span clinical and non-clinical domains, integrating theoretical, empirical, and applied perspectives. Methodologies range from neuroimaging and behavioral studies to systematic reviews, meta analyses, and intervention protocols. Collectively, these studies underscore the versatility of cognitive-motor strategies and their potential for improving functional outcomes, from skill acquisition to neurorehabilitation.

Based on studies examining both the *neurophysiological mechanisms* that support cognitive-motor functions and their *practical applications* in training and therapy, the present collection of articles investigates how action re-enactment can support motor competence, whether in healthy individuals acquiring new skills or in patients recovering lost abilities due to neurological or developmental conditions. Several of these papers explore the integration of these strategies with other modalities, such as motivation-enhancing rewards or traditional physical therapies. This includes randomized controlled trials, pilot studies, systematic reviews, meta analyses, and experimental neuroimaging. Importantly, the research touches on multiple life stages, from children with neurodevelopmental disorders to older adults with degenerative conditions, offering a broad view of how cognitive strategies adapt across contexts.

## Thematic contributions

### Motor learning in non-clinical contexts

While much of the research on cognitive motor strategies is clinical, cognitive motor strategies are increasingly recognized for their role in enhancing skill acquisition in healthy individuals, offering insights into the broader educational and training potential of action re-enactment. Paolini et al. explore this with a novel study on musical skill acquisition. They demonstrate that action observation training (AOT), in which learners observe expert motor actions, can enhance both the accuracy and expressiveness of musical performance in novices. This study broadens the conceptual and applied horizons of AOT, showing its value for fine motor learning in everyday and educational settings.

### Cognitive strategies in stroke rehabilitation

Stroke rehabilitation remains a critical focus for cognitive motor research, where advances in mental simulation techniques and neuroimaging are reshaping how we understand and promote recovery.

Stroke rehabilitation remains a critical application for cognitive strategies. In a robust meta-analysis, Lin et al. evaluate the efficacy of combining action observation and motor imagery (AO + MI) to enhance upper-limb function post-stroke. Their findings reveal consistent and moderate-to-large improvements across randomized controlled trials, positioning AO + MI as a advantageous adjunct to conventional therapy.

Azri et al. complement this with a systematic review of task-based fMRI studies. They reveal how stroke survivors exhibit altered activation patterns, such as compensatory hyperactivation, and demonstrate that these patterns correlate with functional recovery, highlighting the potential of neuroimaging to track and guide rehabilitation.

Finally, Zhao et al. introduce an innovative protocol to assess the impact of reward-based strategies on stroke recovery. Their randomized controlled trial compares fixed vs. probabilistic rewards in motor training, aiming to optimize patient motivation and learning through reinforcement mechanisms.

### Cognitive strategies and pediatric motor impairments

In pediatric populations with neurodevelopmental disorders, understanding the interplay between brain structure and motor cognition is essential for designing age-appropriate and condition-specific interventions.

Understanding motor impairments in neurodevelopmental disorders is a growing area of interest. Martinie et al. investigate feedforward control in children with cerebral palsy (CP), showing that deficits are linked to disrupted white matter tracts, particularly those involved in motor planning. Their results suggest that motor impairments in CP are not solely due to execution deficits but involve broader sensorimotor integration.

Galli et al. provide compelling evidence that children with autism spectrum disorder (ASD) can perform MI tasks comparably to their typically developing peers. These findings challenge strict interpretations of the Broken Mirror Hypothesis and support the feasibility of incorporating MI into therapeutic approaches for ASD.

Ron Baum et al. focus on attention deficit hyperactivity disorder (ADHD), arguing that impairments in motor planning and timing may stem from disruptions in internal motor representations. They advocate for MI as a targeted cognitive intervention to support motor function in affected children.

### Cognitive-motor interventions in aging and neurodegeneration

Older adults and individuals with long-term physical impairments represent a growing target population for innovative action-based therapies that support both functional independence and quality of life.

Martin-Blazquez et al. present a randomized controlled study showing that AOT improves not only motor function but also cognitive outcomes in older adults with mild cognitive impairment (MCI). The intervention enhanced activities of daily living, balance, and cognition, regardless of whether the actions were observed from a therapist or peer.

Joung et al. contribute a pilot study evaluating an adapted dance program for adults with CP. Their findings show improvements in gait parameters and highlight the emotional and physical satisfaction expressed by participants. This points to dance, and possibly other embodied movement therapies, as a promising, enjoyable intervention for adult CP populations.

### Complementary and integrative therapies in neurodegeneration

Li et al. propose a protocol to evaluate the safety and efficacy of traditional Chinese therapies in treating amyotrophic lateral sclerosis (ALS). Though still at the planning stage, this work signals a movement toward integrating culturally diverse, multimodal interventions within the framework of cognitive-motor rehabilitation.

## Conclusions and future directions

The contributions in this Research Topic reveal the remarkable adaptability and effectiveness of cognitive strategies across contexts, from clinical rehabilitation to motor learning. They highlight the mechanisms by which action re-enactment facilitates motor recovery and skill acquisition, and they point toward promising future directions. These include integrating motivational frameworks, exploring home-based and technology-assisted interventions, and advancing standardized methods for cognitive-motor training.

**Figure 1:**
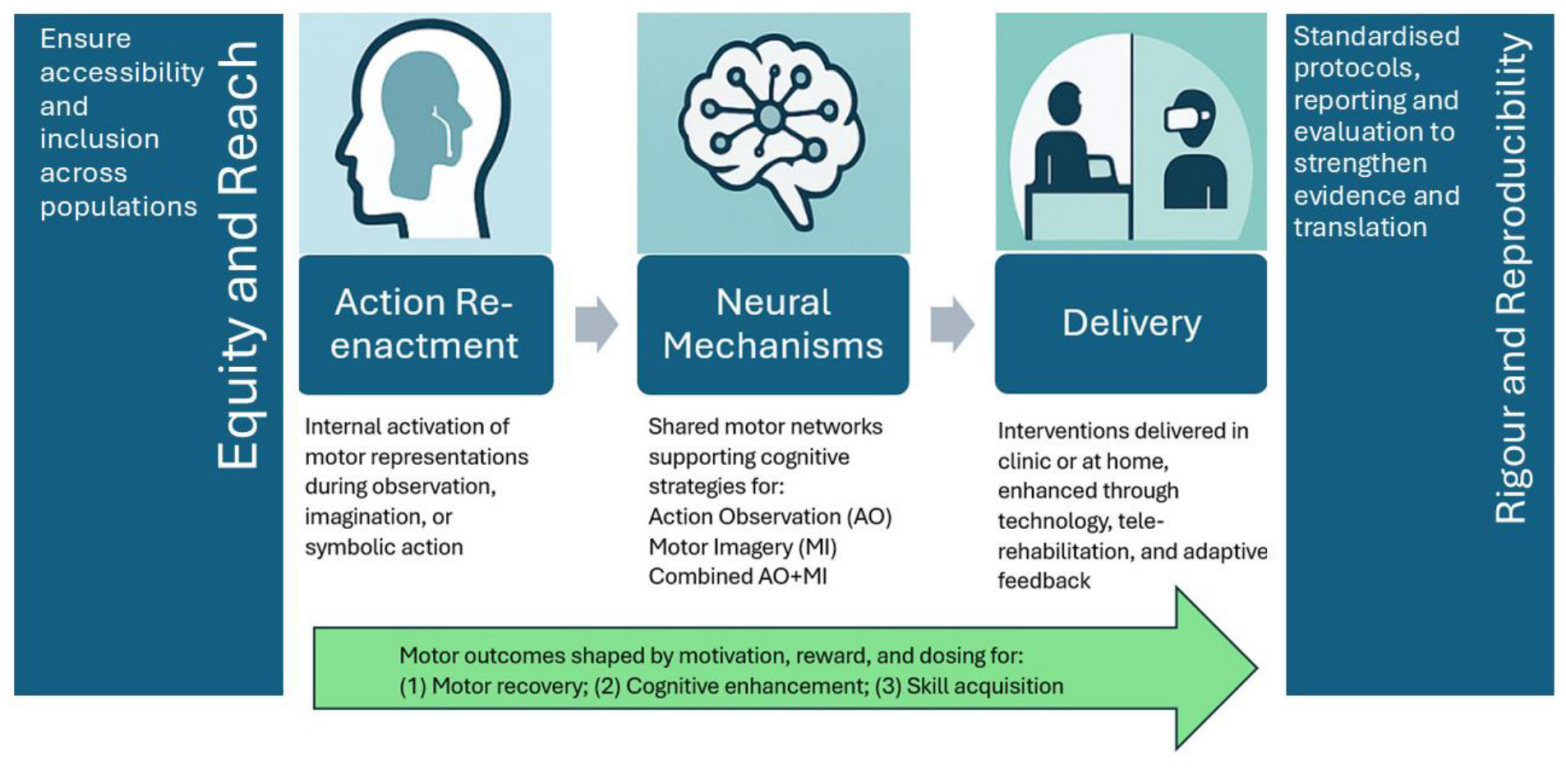
Translational roadmap from action re-enactment to impact. This conceptual framework integrates the key themes of the Research Topic. Action re-enactment provides the foundation for cognitive strategies, Action Observation (AO), Motor Imagery (MI), and their combination (AO + MI), supported by shared neural mechanisms. These strategies translate into applied interventions delivered both in clinical and home-based contexts, increasingly supported by technology. Motivation, reward, and standardized dosing shape their effectiveness, leading to enhanced motor and learning outcomes. Equity and reporting standards flank the translational pipeline, ensuring reach, rigor, and reproducibility across research and practice.

To synthesize the translational pathways highlighted across this Research Topic, we introduce a conceptual “Translational Roadmap from Action Re-Enactment to Impact” (Figure 1). This framework illustrates how neural mechanisms underlying action re-enactment support cognitive strategies such as AO, MI, and AO + MI, which can be implemented through clinic-based and home-based interventions. Motivation, reward, and standardized dosing modulate their effectiveness, driving measurable motor outcomes. The roadmap is framed by principles of equity, rigor, and reproducibility to guide future research and practice toward inclusive and evidence-based translation.

Future research should continue to clarify the neural substrates of action re-enactment, test combinations of interventions (e.g., AO + MI with reward), and extend findings to underserved populations. As this Research Topic illustrates, cognitive strategies grounded in the motor system offer a compelling, scalable path forward for both neuroscience and rehabilitation.

